# Hounsfield units predicts the occurrence but not the patterns of proximal humerus fracture in the elderly patients

**DOI:** 10.1186/s12891-023-06442-6

**Published:** 2023-05-03

**Authors:** Gang Liu, Lu Li, Chengzhi Yang, Lu Wei, Tao Li, Li Zhu, Juzheng Hu

**Affiliations:** 1 Department of Trauma Center, The Fourth Affiliated Hospital of Guangxi Medical University, Liuzhou Worker’s Hospital, Guangxi, 545005 China; 2 Department of Medical Imaging, The Fourth Affiliated Hospital of Guangxi Medical University, Liuzhou Worker’s Hospital, Guangxi, 545005 China

**Keywords:** Computed tomography, Fragility fracture, Hounsfield unit, Neer classification, Proximal humerus fracture

## Abstract

**Background:**

Increased incidence of fragility fractures of the proximal humerus has been reported. Proximal humerus Hounsfield unit (HU) measurements based on computed tomography (CT) scans of the shoulder can be used to evaluate bone mineral density (BMD). It is unknown whether HU values can predict the risk of proximal humerus osteoporotic fracture and /or fracture patterns. Therefore, the objectives of this study were to identify whether the HU value is associated with proximal humeral osteoporotic fracture risk, and whether or not it has an impact on the complexity of the fracture.

**Methods:**

We identified 60 + years old patients’ CT scans between 2019 and 2021 according to the inclusion and exclusion criteria. All patients were divided into two groups based on the presence or lack of a fracture in the proximal humerus, meanwhile, patients with fractures were stratified into simple and comminuted fractures based on the Neer classification. HU values were calculated within the proximal humerus and compared between groups using the Student *t*-test, and receiver operating characteristic (ROC) curve analysis was used to determine the ability of HU values to predict fracture.

**Results:**

A total of 138 patients with proximal humerus fracture (PHF) including 62 simple PHFs and 76 complex PHFs and 138 non-fracture patients were enrolled in the study. The HU values decreased as age increased among all patients. Both male and female patients with PHF had significantly lower HU values compared with non-fracture patients, the area under the curve (AUC) of the ROC curve for males and females was 0.8 and 0.723 respectively. Nevertheless, no significant differences were found between simple and complex fractures of the proximal humerus in the HU values.

**Conclusion:**

Decreasing HU values on CT may be an early warning sign of fracture potential, however, it was not a predictive factor for comminuted fracture of the proximal humerus.

## Introduction

Proximal humerus fractures (PHFs) represent 4–6% of all fractures and are the third most common type of osteoporotic fracture following wrist and hip osteoporotic fractures, which are mainly caused by low-energy trauma and [1]. Some PHFs can be managed conservatively, but displaced unstable PHFs were usually treated surgically such as open reduction internal fixation (ORIF), hemiarthroplasty, and reverse shoulder arthroplasty (RSA) [2, 3]. Surgical treatment choice for PHFs was highly dependent on bone quality, fracture patterns such as degree of displacement, number of fragments, and age. Osteoporotic fractures are associated with low bone mineral density (BMD) and increase in parallel with the aging population. The incidence of osteoporotic fractures varies from 13 to 42% in men to 40–50% in women throughout a human [4]. The occurrence of osteoporotic fractures has generated a huge social and economic impact. Consequently, preventing and predicting osteoporotic fractures should become a major topic in the health and medical fields.

Current evidence suggests that BMD, as measured by dual-energy x-ray absorptiometry (DXA), is the best clinical predictor of osteoporotic fractures. Despite this, the current BMD assessment system is not able to screen peripheral bone quality since DXA only measures the central skeleton. Therefore, a variety of alternative tools have been introduced to evaluate peripheral BMD, including radiographic absorptiometry, ultrasound, and conventional and quantitative computed tomography, particularly, the conventional CT method is one of the most widely used for identifying unrecognized diseases and is easy to [5]. Many studies have evaluated BMD on general CT scans of the spine, hip, and wrist obtained for reasons other than BMD [6–9]. Similarly, three recent literatures compared DXA data with Hounsfield unit (HU) measurements in the proximal humerus, and a significant association between HU values on shoulder CT and BMD as measured by DXA scan was [10–12]. These methods are being studied to identify low BMD and, more importantly, to predict fractures. Studies have compared HU values of distal radius from distal radius fracture patients with controls and found significantly lower regional HU in the fracture [13]. Furthermore, one study has correlated HU values of the distal ulna with fracture incidences and found that the percentage of low HU values in patients with fragility fractures was significantly [14]. However, it is unknown if proximal humerus HU measurement accurately screens for future fragility fractures risk.

The purpose of the present study was to evaluate the local BMD of patients with PHFs and compare the HU values on CT with those of non-fracture patients, and identify whether the HU values would be predictive of PHF risk. In addition, we hypothesized that bone quality influences the complexity of PHFs, and low HU values of the proximal humerus meant poor bone quality, 3- and 4-part fractures are more complex and might have lower bone quality compared to 1- and 2-part fractures.

## Materials and methods

### Study cohort

A retrospective case-control study was conducted among 60 years or older patients, and it was approved by the ethics committee of our hospital, informed consent was waived because of the retrospective nature of this study. 138 patients with proximal humerus fractures (PHFs) due to low-energy trauma were included for evaluation between 2019 and 2021. In the fracture cohort, CT data were acquired within one week of injury to minimize the effect of disuse osteoporosis. To investigate the study question equally between the sexes, convenience sampling was used to select the same numbers of male and female patients. A picture-archiving communication system (PACS) database search was used to determine 138 age and sex-matched control patients who visited the hospital for routine physical examination and had a chest CT scan including both shoulders on which no fracture was identified. For both cohorts, patients with any co-existing diseases affecting bone quality(such as osteoarthritis, rheumatoid arthritis, infectious arthritis and gouty arthritis of the shoulder, frozen shoulder, shoulder instability, and rotator cuff injury) and pathologic fractures were excluded. As the Neer classification [15] is widely used for the assessment of PHFs, we employed it to describe the fracture cohorts in this study, and the PHFs were divided into two subgroups: simple fractures (1- and 2-part fractures) and comminuted fractures (3- and 4-part fractures) according to it.

### Hounsfield Unit Methodology

**Fig. 1:**
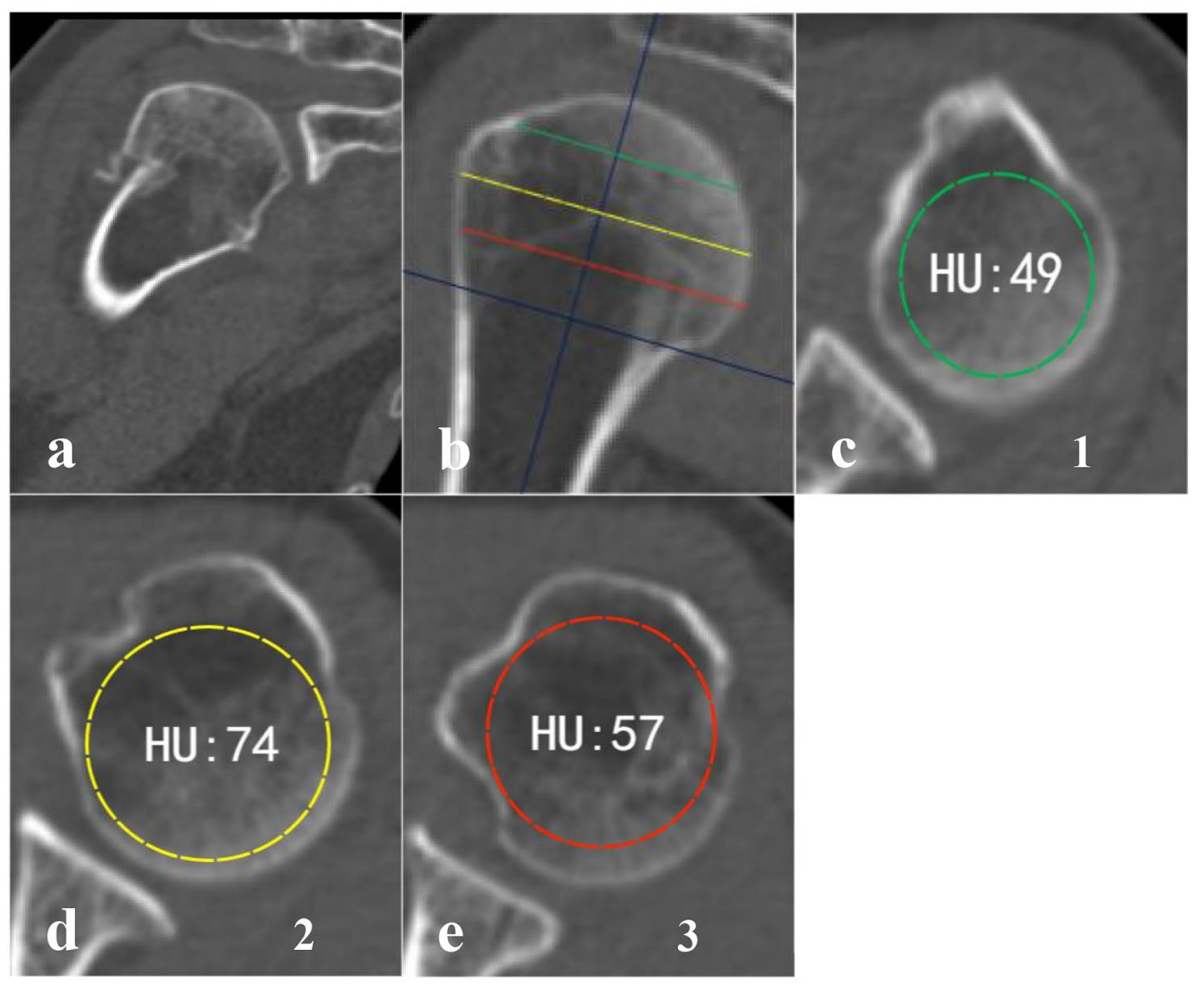
Method of measuring the proximal humerus average HU value. CT image of the patient with proximal humerus fracture(a). CT image of the same patient, the contralateral uninjured proximal humerus. The center of the humerus medullary cavity and the height of the head were determined first. Then, three axial planes equidistant to each other were defined(b). ROIs were placed in the humerus head in each of the previously defined axial planes(c-e). CT, computed tomography; ROI, region of interest

Patients were placed in the supine position with their shoulders in the neutral position. CT images were acquired with a 32-slice multidetector spiral CT (SIEMENS, Germany) with the following parameters: 120 kV; 350 mA; 1500 HU (window width); 300 HU (window level); 1.0 mm (scanning layer thickness); and 0.6 mm (scanning interval). Bone mineral density (BMD) between left and right humeri revealed a high correlation for the proximal [16], thereby the HU values of the healthy proximal humerus were measured based on the method described by Pervaiz et [17]. Briefly, the HU values were determined by performing standardized measurements in the reconstructed sagittal plane, the distance between the superior edge of the humerus head and approximately the surgical neck along the midline was quartered, and three equidistant axial planes were defined. PACS software was used to calculate HU values within the proximal humerus after switching to the horizontal image, all elliptical regions of interest (ROIs) were isolated to cancellous regions of bone, with avoidance of cortical regions (Fig. 1). Following this procedure, HU values from the three axial slices were averaged to generate an average HU value for each sample and the mean HU values were used in the final analysis. All measurements were made by two independent orthopaedic surgeons.

### Statistical analysis

Statistical analysis was performed using SPSS (IBM SPSS 23.0, SPSS Inc). Continuous variables were presented as means and standard deviation (SD) and compared using paired Student *t*-test or unpaired Student *t*-test. To assess the relationship between age and HU values in the non-fracture cohort, we calculated the Pearson R coefficient with a two-tailed *p*-value. Standard receiver operating characteristic curve (ROC) analysis was performed to determine the ability of HU values to discriminate fracture status. *p*-values less than 0.05 (*p* < 0.05) were considered significant.

## Results

In this retrospective study, 138 patients with proximal humerus fractures (PHFs) and identical numbers of male and female non-fracture patients fulfilled the inclusion criteria without meeting the exclusion criteria. The fracture and control cohorts included 52 males and 86 females respectively and each group was similar in age and sex (Table 1). According to the Neer classification, there were 61 patients with simple PHFs (15 Neer I fractures and 46 Neer II fractures) and 77 patients with complex PHFs (46 Neer III fractures and 31 Neer IV fractures) in the fracture cohorts (Table 2).

**Fig. 2:**
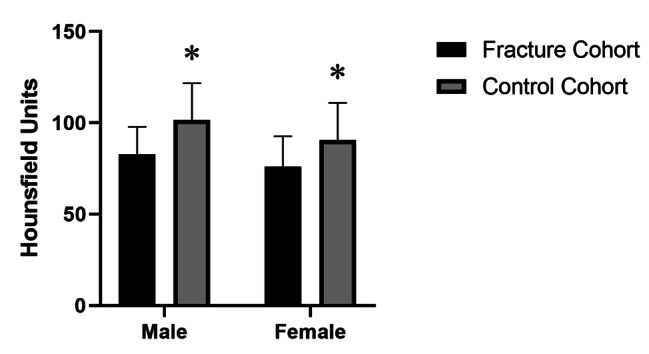
A comparison of the mean Hounsfield unit values in the proximal humerus fracture cohort and the control cohort. Difference between the two cohorts was significant for both sexes (*p＜0.001)

**Fig. 3:**
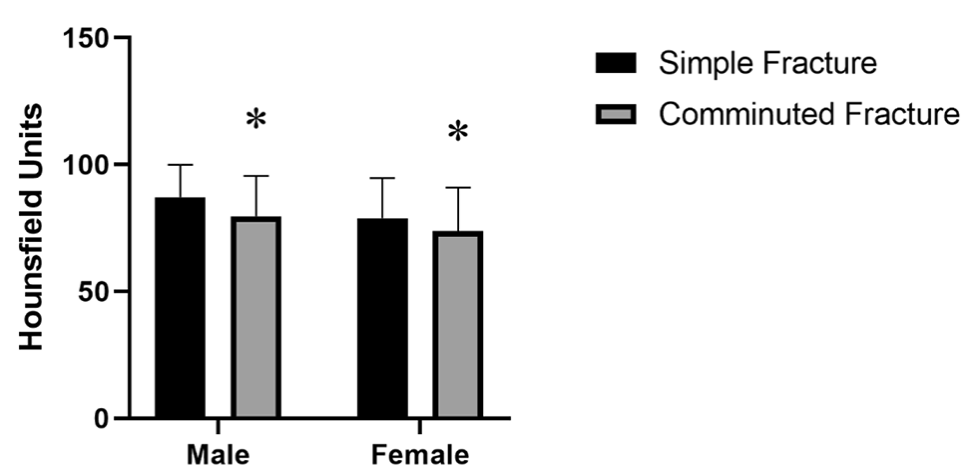
A comparison of the mean Hounsfield unit values in the simple fractures and the comminuted fractures. There was no statistical difference between the two groups in both sexes (*p＞0.05)

Compared to age-matched non-fracture control patients, both male and female patients with PHFs had significantly lower local HU values as measured by CT (*p* < 0.01) (Fig. 2). The HU values of proximal humerus showed no significant difference between patients with simple and comminuted fracture patterns (*p*＞0.05) (Fig. 3).

**Fig. 4:**
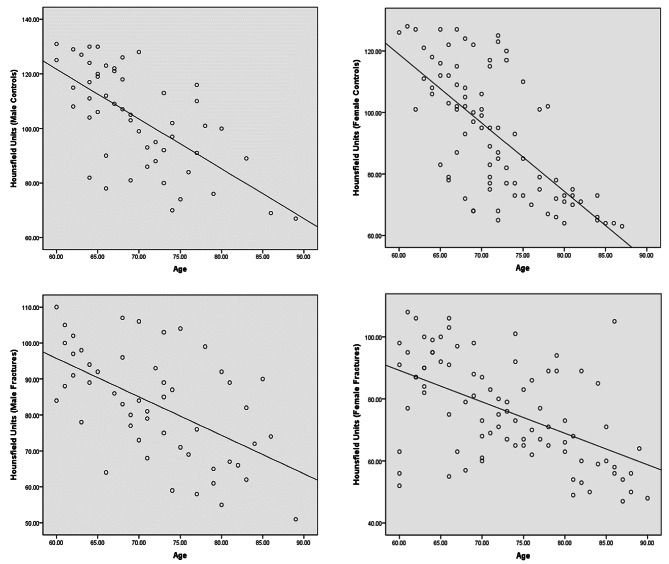
An analysis of the correlation between patient age and HU values of the proximal humerus, as assessed by Hounsfield unit measurements. A significant decrease in HU values with increasing age was observed in all patients of both sexes

For females, the area under the curve (AUC) for the receiver operating characteristic curve (ROC) for identifying fractures based on the HU values was 0.723 (95% CI, 0.649–0.798), an HU value of 100 for the proximal humerus optimized sensitivity (93%) and specificity (42%) for distinguishing fracture patients from controls. For males, the AUC was 0.8 (95% CI, 0.717–0.884), with an HU value of 98 optimized sensitivity (83%) and specificity (64%) (Fig. 4).

**Fig. 5:**
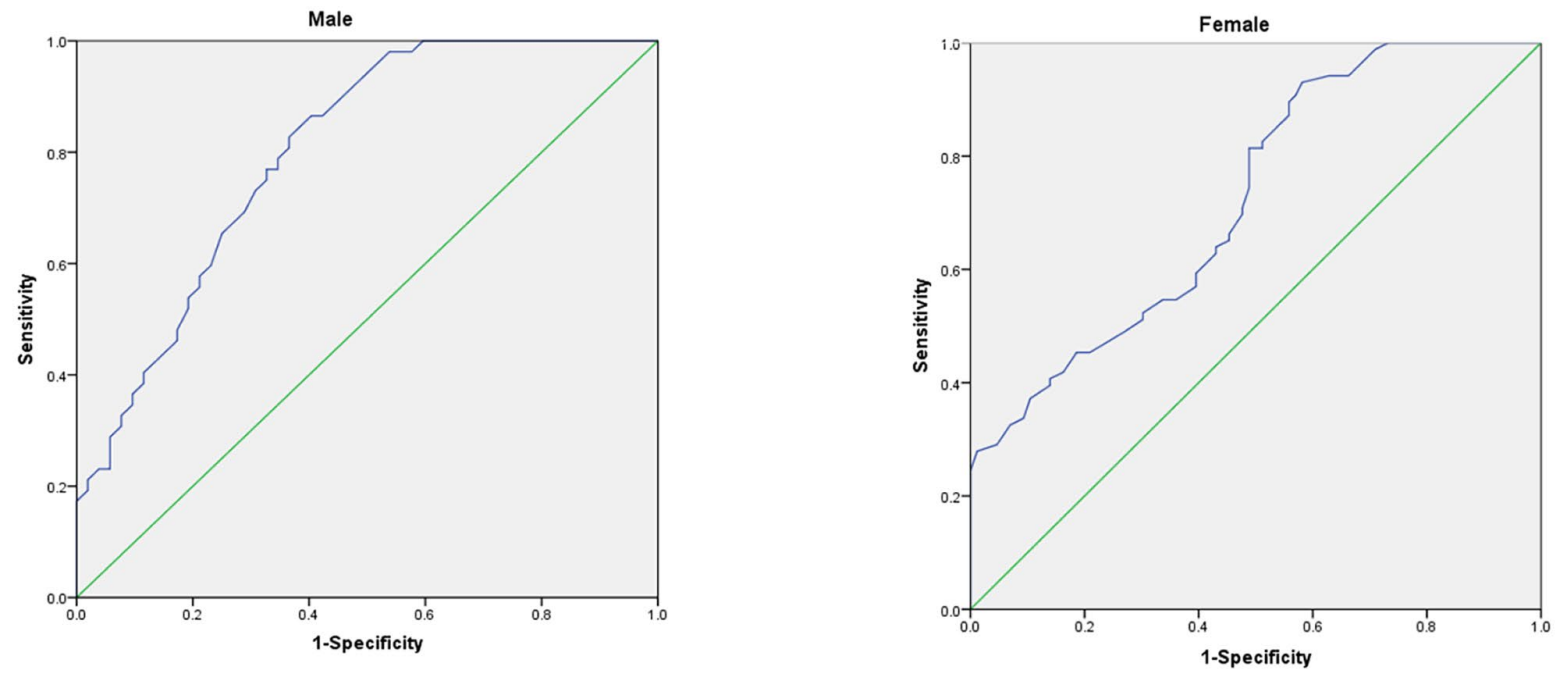
Receiver operating characteristic (ROC) curve to identify fracture based upon HU value

In the control cohorts, both male (r = 0.65; *p* < 0.01) and female (r = 0.689; *p* < 0.01) patients showed an age-related decline in proximal humerus HU values, and there were lesser correlations between age and HU values among the fracture cohorts, as male (r = 0.553; *p* < 0.01) and female (r = 0.524; *p* < 0.01) (Fig. 5).

**Table 1:** Patient Characteristics*

	Control Cohort	Fracture Cohort
Male age (yr)		
Mean	69.9	72.1
Range	60–89	60–89
Female age (yr)		
Mean	71.8	72.9
Range	60–87	60–90

*N = 138 in each cohort (52 male and 86 female patients).

**Table 2:** Neer classification for patients with proximal humeral fractures

	Number of cases	Neer Classification			
		I	II	III	IV
Male	52	5	17	19	11
Female	86	10	29	27	20

## Discussion

As the population ages, the incidence of osteoporosis-related fractures in the elderly increases every year. It is estimated that one osteoporotic fracture occurs every 3 s on average and osteoporosis causes more than 8.9 million fractures, [18]. Following hip fractures and distal radial fractures, proximal humerus fractures (PHFs) are the third most common fractures in elderly patients, and the PHFs independently increased the risk of future fracture several times in the first year on an individual [19, 20]. This suggests that fragility fracture can concomitantly generate financial and health burdens in elderly individuals, thus, a quick, easy, and efficient osteoporosis/fracture screening tool may be useful for optimizing appropriate management.

Bone mineral density (BMD) measurement by dual-energy X-ray absorptiometry (DXA) and peripheral quantitative computed tomography (CT) has been commonly used to evaluate the risk of osteoporosis owing to a variety of causes, and associated [21]. Despite that, those screening tool is not without their failings. DXA of the lumbar vertebral body and proximal femur is routinely used in the diagnosis/screening of osteoporosis in the clinical setting, which represents only the BMD of the central skeleton, is limited for reflecting the quality and the fracture risk of upper extremity [22], such as proximal humerus.

Studies have been conducted to assess the local BMD in the proximal humerus, and it has been demonstrated that cortical index (CI), deltoid tubercle index (DTI), and Hounsfield unit (HU) of the proximal humerus are associated with local bone [10, 11, 17, 21, 23–25]. In clinical practice, the measurement of CI and DTI requires a standardized position (patients suffering from pain after sustaining a PHF often lack an ideal projection in the AP view), and radiography quality is affected by the scanning parameters, furthermore, a significantly reduced cortical bone thickness as a result of osteoporosis makes it difficult to accurately identify the inner margin of cortical bone in the proximal [26, 27]. Therefore, these factors may ultimately influence the accuracy of bone quality. The HU values derived from CT scanning express the local BMD accurately and are not affected by human [28], and a study had shown that bone quality at the proximal humeral is best predicted by BMD measurements at the contralateral [16]. Therefore, we measured the HU values of the contralateral proximal humerus. The intra- and interobserver reliability of the measurement of HU values was very [10, 12, 17] so we did not further validated them in the present study.

In this study, we found that both male and female patients with a PHF had lower local bone quality, as assessed by HU measurements, in the proximal humerus (Fig. 5), and a high correlation between age and HU values (Fig. 2). It is consistent with previous [10]. Currently, DXA is the gold standard for osteoporosis diagnosis, as well as an effective tool for predicting fractures. DXA scanning was described by Doetsch as a way to measure the local BMD of the proximal humerus [29]. HU values measurement based on CT have been used clinically to assess fracture risk and numerous studies have suggested that lower HU values are a predictive factor for subsequent fragility [13, 14, 28, 30]. In the present study, The HU values of the proximal humerus had a higher area under the curve (AUC) for the diagnosis of PHFs and had a higher sensitivity. Moreover, we established the proximal humerus HU value cut-off that may be used as guidelines for alerting the physician to potential osteoporosis or fracture. According to our results, we suggest that males with a proximal humerus HU value of < 98 and females with a value of < 100 be considered for further BMD screening, and below our predetermined cut-off values, may be more likely to suffer a future fragility fracture.

As of yet, no clear criteria or reasons had been provided for diagnosing a severe PHF, a review of the literature found that much of the literature referring to severe PHF was based on the Neer classification [31–33]. In the present study, low-energy PHF in patients aged over 60 were classified according to the Neer classification, 1- and 2-part fracture was defined as simple PHF while 3- or 4-part fracture was defined as comminuted PHF. We investigated the correlation between the severity of PHFs and local bone quality based on HU values in the proximal humerus. Contrary to our hypothesis, no significant difference was observed between simple and complex fractures of the proximal humerus in HU values (Fig. 3). Thereby, the result indicated that the complexity of a PHF depends on other factors than the local HU values measured by CT scanning. A study has been conducted to assess the parameters of bone microarchitecture and cortical index and to compare these parameters with the severity of fractures, and found that there was a considerable difference in the mechanisms and intensities of trauma that caused fractures between simple and complex fractures [34], suggested the mechanisms of trauma may have a greater effect on fracture severity than the local bone structure. A previous [35] proved a clear correlation between the BMD of the hip and the severity of distal radial fractures, while another recent [36] did not find the same association between the BMD and PHFs, interestingly, patients with complex fractures had significantly higher body mass index(BMI), this is investigated in another paper which confirmed that BMI was significantly higher in the complex fracture cohort based on the AO/OTA [37]. Similar findings were also revealed in the present study. The present study found that, as age increases by one year, the incidence of severe PHF increases by 1.064 times, this result is comparable to the results (1.044 times) of Kim et al.[38], additionally, they indicated that gender was the independent risk factor of severe PHFs considering that females were 3.763 times more likely to suffer from severe PHF than males, without a clear cause. This study only included patients greater than or equal to 60 years old, here we did not find a similar result. Further population-based research is needed to determine the clear cause of this finding.

There are limitations within the present study. In this study, the HU values were not correlated with BMD measured by DXA due to the lack of equipment in emergency units and standardized position in the acute setting. BMI was not taken into account, because height or weight was not recorded in all patients, and sarcopenia and nutritional status were not assessed, those factors might affect the results of this study. Despite the present study’s identified HU value cut-off, additional research is required to prove correlations between proximal humerus DXA values and HU values before the method can be truly diagnostic. Additionally, we were unable to show a difference in the rate of future fragility fractures between the PHF cohorts and control cohorts because of the retrospective study. Further investigations should be taken into account in future large prospective cohort studies to elucidate those questions.In conclusion, our study population showed an age-related decline in proximal humerus BMD, many more patients may fracture with age, highlighting the value of early screening and intervention. This novel method requires no additional testing or manipulation of the images and can potentially quickly and accurately detect patients at risk for a PHF based on the HU values derived from conventional CT scans. Unfortunately, local HU values based on HU values measured by CT could not predict the severity of PHFs in the elderly, and other factors, such as age, BMI and hormone, etc. seem to have a great impact on the occurrence of more complex fractures. Further studies with larger numbers of cases and a wider range of ages are required to confirm our data.

## Data Availability

The datasets used or analyzed during the current study are available from the corresponding author on reasonable request.
